# Study on Lifetime Performance Evaluation of a Precast Prestressed Concrete Frame in Chloride Environments

**DOI:** 10.3390/ma16206666

**Published:** 2023-10-12

**Authors:** Jun Yang, Zhaoming Yuan, Jie Liu, Shuqi Yu

**Affiliations:** 1 Jiangsu Key Laboratory of Structure Engineering, Suzhou University of Science and Technology, Suzhou 215011, China; yzm13775443626@163.com; 2 School of Civil Engineering, Southeast University, Nanjing 211189, China; yushuqi@seu.edu.cn; 3 College of Civil Engineering, Nanjing Forestry University, Nanjing 210037, China; jieliu@outlook.com

**Keywords:** precast prestressed concrete frame, corrosion, fragility analysis, time-varying seismic performance, lifetime performance evaluation

## Abstract

This study established a comprehensive framework for evaluating the lifetime performance of precast prestressed concrete frames exposed to chloride environments. The proposed analytical framework enabled a scientifically grounded and rational assessment of both the service life and residual load-carrying capacity of precast prestressed concrete frames in chloride environments. It further served as the foundational basis for making informed decisions regarding the repair and maintenance of pertinent structures. Based on Fick’s second law, this evaluation framework established the probability distribution of the corrosion initiation time and cracking time of reinforced concrete structures due to corrosion expansion in a chloride environment. Additionally, based on the fragility analysis model and results of a precast prestressed concrete frame in a chloride environment, a practical method for evaluating the time-varying seismic performance of the precast structure considering the influence of corrosion was proposed. Furthermore, by employing the path probability model and reliability theory, time-varying reliability models were proposed to predict the three limit states of the precast prestressed concrete frame. According to the analysis results of a four-story planar frame, it could be seen that the corrosion initiation time and normal service limit state were highly sensitive to the chloride ion diffusion coefficient of the composite layer in precast concrete structures. Compared to cast-in-place structures, the presence of a composite layer in precast concrete structures could lead to more severe degradation of the time-varying seismic performance of the precast prestressed concrete frame.

## 1. Introduction

The corrosion of reinforced concrete structures leads to a notable decline in the mechanical properties of the reinforcing steel, as well as a reduction in the bond strength between the reinforcement and concrete. In recent years, researchers worldwide have conducted extensive experimental investigations into the mechanical properties of corroded steel reinforcement [1,2,3]. The results indicate a significant deterioration in the mechanical properties of steel with increasing degrees of corrosion. Numerous scholars have systematically investigated the influence of corrosion on the bond-slip behavior between the reinforcement and concrete, and have proposed corresponding degradation models based on characteristic parameters [4,5,6,7].

Rebar corrosion leads to a reduction in the cross-sectional area and mechanical property degradation, resulting in a deterioration of the steel–concrete bond strength, as well as the occurrence of cracking and potential spalling of the concrete cover. These combined effects contribute to a degradation in the seismic performance and a shift in the failure modes of the structure. Yang et al. [8,9] conducted a comprehensive study on the seismic performance of interior and exterior beam–column joints in a precast prestressed concrete frame subjected to chloride erosion. The results indicated that a progressive deterioration in the seismic performance of the beam–column joints with an increase in the corrosion ratio, potentially leading to a change in the failure mode of the structure. Zhang and Li [10] conducted a study on the seismic performance of corroded reinforced concrete exterior joints. The results showed that the corrosion of reinforcements had a significant adverse effect on the strength and lateral drift capacity of the joints as well as other mechanical properties.

Presently, experimental studies on corroded concrete structures often concentrate on specimens with specific corrosion ratios, potentially limiting the representation of the structure’s complete lifetime degradation process. Additionally, due to the considerable uncertainty associated with seismic forces, employing simple cyclic loading may not offer a comprehensive assessment of the structure’s seismic performance. Consequently, scholars have introduced seismic vulnerability analysis methods to provide a holistic evaluation of structural seismic performance [11]. Choe et al. [12] established a seismic vulnerability analysis model for corroded steel reinforcement concrete columns by considering material uncertainty and structural geometric uncertainty. In a similar vein, Liu et al. [13] conducted a study on the time-dependent seismic fragility of typical concrete girder bridges subjected to chloride-induced corrosion. Their findings underscored the significance of considering the collective effects of various deterioration mechanisms when modeling the time-dependent seismic fragility of aging bridge systems.

Some scholars have conducted research on prediction methods for corroded materials or structures using statistical or machine learning approaches. Weyers proposed a model to estimate the service life of corrosion protection systems, and the model consisted of three serial phases [14]. Darmawan introduced statistical parameters derived from accelerated corrosion tests to analyze the maximum pit depth distribution of corroded steel in reinforced concrete structures. By utilizing these parameters, one can predict the service life of reinforced concrete structures in chloride-rich environments [15]. Naser established a framework for integrating explainable and anomalous machine learning into a digital twin, enabling fine-tuning of mixtures as a means to develop next-generation concrete with enhanced performance. Furthermore, this framework exhibits scalability across multiple platforms [16]. These methods could serve as valuable references for evaluating the lifetime performance of precast prestressed concrete frames in a chloride environment.

Over the past decades, research on the seismic performance of precast prestressed concrete frame structures has been conducted worldwide, including theoretical analyses and cyclic loading/vibration table tests [17,18,19,20]. It is noteworthy that these structures have found applications in coastal environments. For precast concrete structures, the presence of post-pour overlap layers (composite layer) renders them more susceptible to infiltration by corrosion mediums, exacerbating durability concerns. However, research on the comprehensive lifetime performance assessment of precast concrete structures in chloride environments remains limited. Therefore, this paper established a comprehensive framework for evaluating the lifetime performance evaluation of precast prestressed concrete frame structures in chloride environments. The goal is to provide a scientific and rational assessment of the structure’s remaining load-carrying capacity, offering valuable insights into pertinent structural maintenance and reinforcement decision-making.

## 2. Lifetime Assessment Framework

**Figure 1:**
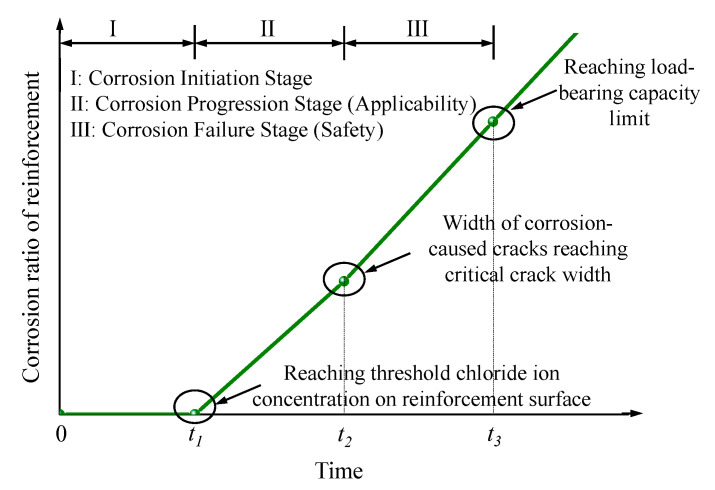
Time-dependent characteristics of structural performance in chloride environments.

The corrosion of steel reinforcement is one of the significant factors leading to the degradation of concrete structure performance [21]. Figure 1 illustrates the process of performance degradation in conventionally cast-in-place reinforced concrete structures due to chloride ion erosion [22,23]. Upon comparative analysis of the degradation mechanisms and stages between cast-in-place and precast concrete structures, it becomes evident that no significant differences emerge. Therefore, the performance degradation process depicted in Figure 1 can be equally applied to the analysis of precast concrete structures.

**Figure 2:**
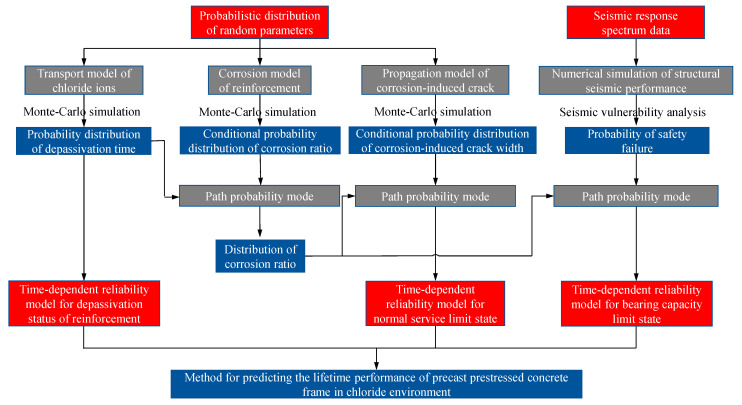
Lifetime assessment framework of the precast prestressed concrete frame.

To construct a time-dependent reliability analysis model based on reliability theory, it is imperative to initially ascertain the time-varying probability models for the structure at each corrosion stage. Subsequently, predictive time-varying models for the three limit states of precast prestressed concrete frame structures can be established using path probability models. These predictive time-varying models for the three limit states encompass time-varying reliability models for the rebar depassivation state, time-varying reliability models for the normal service limit state, and time-varying reliability models for the load-bearing capacity limit state under varying performance level requirements. The specific framework is illustrated in Figure 2.

## 3. Reliability Model

When employing probabilistic statistical methods for the design calculation of the lifetime of a structure, the structural performance function, denoted as *Z*, is defined as the difference between the resistance *R* against “failure” and the environmental load *F*, i.e., *Z* = *R* − *F*. The probability of structural failure *P_f_* is defined as the maximum target probability *P_target_*, and is represented as follows:

(1) $$P_{f}=P(Z<0)<P_{target}$$

Assuming the probability density function of *F* is *f_F_*(*f*) and the cumulative distribution function is *F_F_*(*f*), the probability density function of *R* is *f_R_*(*r*), and the cumulative distribution function is *F_R_*(*r*). If the variables *F* and *R* are independent of each other, then the probability of structural failure *P_f_* is:

(2) $$\begin{array}{ll} P_{f} & =P(R-F<0)=P(Z<0)\\ & =\iint_{r<f}f_{R}(r)\cdot f_{F}(f)drdf\\ & =\int_{0}^{+\infty }\left[\int_{0}^{f}f_{R}(r)dr\right]\cdot f_{F}(f)df\\ & =\int_{0}^{+\infty }F_{R}(r)\cdot f_{F}(f)df \end{array}$$

Or,

(3) $$\begin{array}{ll} P_{f} & =P(R-F<0)=P(Z<0)\\ & =\iint_{r<f}f_{R}(r)\cdot f_{F}(f)drdf\\ & =\int_{0}^{+\infty }\left[\int_{f}^{+\infty }f_{F}(f)df\right]\cdot f_{R}(r)dr\\ & =\int_{0}^{+\infty }\left[1-F_{F}(f)\right]\cdot f_{R}(r)dr \end{array}$$

For a normal distribution, the failure probability can be expressed as:

(4) $$P_{f}=\Phi (-\beta )\frac{-b\pm \sqrt{b^{2}-4ac}}{2a}$$

where *β* is the reliability index, indicating the acceptable level of failure for various limit states of the structure.

According to the aforementioned definition, when calculating the service life of a concrete structure at the corrosion initiation stage, the state of “failure” is defined as the chloride ion concentration (*c*(*x*)) reaching the critical concentration that induces rebar corrosion at the depth of the protective layer. In other words, the surface chloride ion concentration on the rebar reaches a threshold (*c_cr_*). Consequently, the resistance *R* against this “failure” is represented by the thickness of the concrete protective layer *x*. The environmental load serves as the driving force for chloride ion migration within the concrete. Therefore, the probability of structural failure *P_f_* in this phase can be expressed as:

(5) $$P_{f}=P[(c(x)-c_{cr})<0]$$

At the corrosion propagation stage, if the critical crack width (*w_cr_*) on the surface of the component is used as the criterion for structural failure, the probability of structural failure *P_f_* can be expressed as:

(6) $$P_{f}=P\left[(w-w_{cr})<0\right]$$

For different limit states, there are certain differences in the reliability index, as shown in Table 1.

At the corrosion failure stage, the probability of structural failure varies due to different requirements for structural performance levels. In this phase, a time-dependent probability model for the ultimate limit state of bearing capacity will be established based on Equation (7). *IM* represents the seismic motion parameters, *EDP* represents the structural engineering demand parameters, and *C* represents the structural seismic capacity parameters. Under different seismic actions and corrosion levels, the structural response is highly stochastic. Therefore, the seismic vulnerability analysis method based on incremental dynamic analysis (IDA) will be employed to formulate a time-dependent reliability model for the ultimate limit state of bearing capacity, tailored to the varying performance level requirements during the corrosion failure stage.

(7) $$P_{f}=P(EDP>C\left|IM=x\right.)$$

## 4. Time-Dependent Probability Models for Each Stage

According to Equations (2) and (3), it can be observed that the limit state equation for concrete durability assessment in a chloride environment involves highly nonlinear and complex partial derivatives, making the solution of high-order equations a formidable task. The application of direct integration methods is particularly challenging in this context. Currently, the Monte-Carlo simulation is one of the most effective methods for conducting reliability assessments of complex structures.

### 4.1. Corrosion Initiation Stage

The research suggests that, among various chloride ion erosion mechanisms, diffusion plays a notably more significant role. The diffusion of chloride ions in concrete is commonly modeled by Fick’s second law [21,26]. Collepardi et al. [27] developed a chloride ion diffusion model based on Fick’s second law. The concentration of chloride ions at any given time (*t*) and depth (*x*) from the concrete surface can be calculated using Equation (8).

(8) $$C(x,t)=C_{s}\left[1-erf\left(\frac{x}{2\sqrt{Dt}}\right)\right]$$

where *C_s_* represents the surface concentration of chloride ions in concrete, *erf*(⋅) stands for the error formula, and *D* denotes the diffusion coefficient of chloride ions.

Based on the classical diffusion theory, the Duracrete model [28] not only incorporates the time-dependent aspects of chloride ion diffusion but also comprehensively addresses the impacts of factors such as material properties and external environmental conditions, as shown in Equation (9).

(9) $$C\left(x,t\right)=C_{s}\left[1-erf\left(\frac{x}{2\sqrt{k_{e}k_{l}k_{c}D_{0}{\left(t_{0}\right)}^{n}{\left(t\right)}^{1-n}}}\right)\right]$$

where *k_e_*, *k_l_*, and *k_c_* represent the modification coefficients for the environmental influence, the testing method, and the curing conditions, respectively. *D*_0_ stands for the reference chloride ion diffusion coefficient at a concrete age of *t*_0_.

### 4.2. Corrosion Progression Stage

Chloride ions continuously infiltrate into concrete, gradually accumulating on the surface of the steel bars. When the chloride ion concentration on the surface of the steel bars reaches a critical level, corrosion of the steel bars initiates. This signifies the onset of the corrosion progression stage, which persists until corrosion-induced cracks ultimately reaches the critical crack width. This denotes the end of the corrosion progression stage. Generally, steel corrosion includes two forms: uniform corrosion and non-uniform corrosion [29,30,31].

Uniform corrosion leads to a reduction in the diameter of the steel bar. At time *t* after corrosion initiation, the remaining cross-sectional area of the steel bar can be determined using Equations (10)–(12).

(10) $$A\left(t\right)=\frac{\pi d_{s}{\left(t\right)}^{2}}{4}$$

(11) $$d_{s}\left(t\right)=\left\{\begin{array}{cc} d_{s0} & t\leq T_{cor}\\ d_{s0}-2\lambda \left(t-T_{cor}\right) & T_{cor}<t\leq T_{end}\\ 0 & t>T_{end} \end{array}\right.$$

(12) $$T_{end}=\frac{d_{s0}}{2\lambda }+T_{cor}$$

where *d_s_*(*t*) represents the remaining diameter of the reinforcing bar at time *t*, *d_s_*_0_ is the initial diameter of the reinforcing bar, *λ* is the corrosion rate of the reinforcing bar, *T_cor_* is the start time of the corrosion of the reinforcing bar, and *T_end_* is the end time of the corrosion of the reinforcing bar.

Because of the generation of corrosion current during the corrosion process for the reinforcing bars, most corrosion models are predicated on the corrosion current density (*i_cor_*). Generally, the average corrosion rate of the reinforcing bars can be determined using Equation (13) [31].

(13) $$\lambda =0.0116i_{cor}\left(t\right)$$

Sung et al. [32] proposed Equation (14) to calculate the corrosion rate of reinforcing bars after the occurrence of corrosion cracking in the protective layer.

(14) $$\lambda =2.5\times 0.0116i_{cor}\left(t\right)$$

where *i_cor_*(*t*) represents the corrosion current density at time *t*.

Based on the experimental results of Liu et al. [33], Vu et al. [34] proposed empirical formulas for the variation of the corrosion current density in reinforcing bars with specimen changes, as shown in Equations (15) and (16).

(15) $$i_{cor}\left(t\right)=0.85\times i_{cor,0}{\left(t-T_{cor}\right)}^{-0.29}$$

(16) $$i_{cor,0}=\frac{37.8{\left(1-w/c\right)}^{-1.64}}{x}$$

where *i_cor,_*_0_ represents the corrosion current density at the beginning of corrosion, and *w/c* stands for the water–cement ratio.

**Figure 3:**
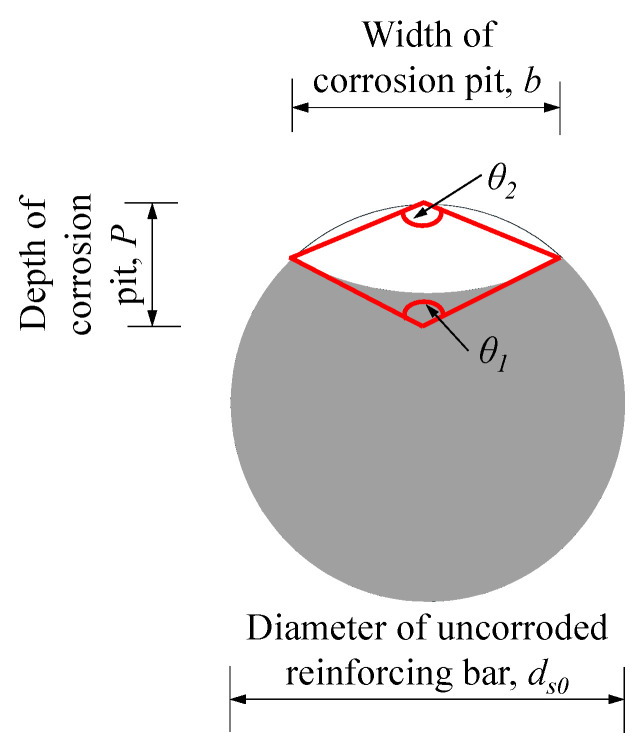
Corrosion pit diagram.

Under the erosion of chloride ions, steel bars will also undergo non-uniform corrosion (local pitting), which first leads to the appearance of corrosion expansion cracks in the concrete at the site of severe non-uniform corrosion. According to the investigation by Val et al. [35], the geometric shape of pitting can be approximately simplified as a quadrilateral, as shown in Figure 3. This simplified shape can be used to predict the pitting area. The specific calculation is performed using Equations (17)–(21).

(17) $$\Delta A_{s}=\left\{\begin{array}{cc} A_{1}+A_{2} & \;P(t)\leq \frac{d_{s0}}{\sqrt{2}}\\ \frac{\pi d_{s0}^{2}}{4}-A_{1}+A_{2} & \frac{d_{s0}}{\sqrt{2}}<P(t)\\ \frac{\pi d_{s0}^{2}}{4} & \;P(t)\geq d_{s0} \end{array}\right.\leq d_{s0}$$

(18) $$\left\{\begin{array}{c} A_{1}=\frac{1}{2}\left[\theta_{1}{\left(\frac{d_{s0}}{2}\right)}^{2}-b\left|\frac{d_{s0}}{2}-\frac{P{\left(t\right)}^{2}}{d_{s0}}\right|\right]\\ A_{2}=\frac{1}{2}\left[\theta_{2}P{\left(t\right)}^{2}-\frac{bP{\left(t\right)}^{2}}{d_{s0}}\right] \end{array}\right.$$

(19) $$\left\{\begin{array}{c} \theta_{1}=2\arcsin\left(\frac{b}{d_{s0}}\right)\\ \theta_{2}=2\arcsin\left(\frac{b}{2P(t)}\right) \end{array}\right.$$

(20) $$b=2P\left(t\right)\sqrt{1-{\left(\frac{P(t)}{d_{s0}}\right)}^{2}}$$

where *P*(*t*) represents the depth of the pitting corrosion, typically calculated by adjusting the uniform corrosion depth, as shown in Equation (21).

(21) $$P\left(t\right)=R\lambda$$

This study employs the model provided in reference [36] to predict the crack width, as shown in Equations (22) and (23).

(22) $$\omega =K\left(\Delta A_{s}-\Delta A_{s0}\right)$$

(23) $$\Delta A_{s0}=A_{s}\left[1-{\left[1-\frac{\alpha }{d_{s0}}\left(7.53+9.32\frac{x}{d_{s0}}\right)10^{-3}\right]}^{2}\right]$$

where *ω* represents the crack width (mm) and *K* is the fitting parameter.

### 4.3. Corrosion Failure Stage

Relying solely on pseudo-static tests and related numerical simulations may not comprehensively reflect the response of structures under seismic action. Therefore, this study employs the incremental dynamic analysis (IDA) method to conduct nonlinear dynamic time history analysis on prestressed concrete-assembled integral frames with varying degrees of corrosion. By statistically analyzing the IDA data, seismic vulnerability curves for the structures are derived, allowing for the assessment of the time-dependent seismic performance of prestressed concrete-assembled integral frames.

This study, referencing [37], employs S_a_(T1, 5%) as the seismic intensity indicator and utilizes the maximum inter-story drift angle (*θ_max_*) as the structural damage indicator. In accordance with [38,39], four performance levels have been selected: operational (OP, *θ_max_* = 0.5%), immediate occupancy (IO, *θ_max_* = 1.0%), life safety (LS, *θ_max_* = 2.0%), and collapse prevention (CP, *θ_max_* = 0.5%).

**Figure 4:**
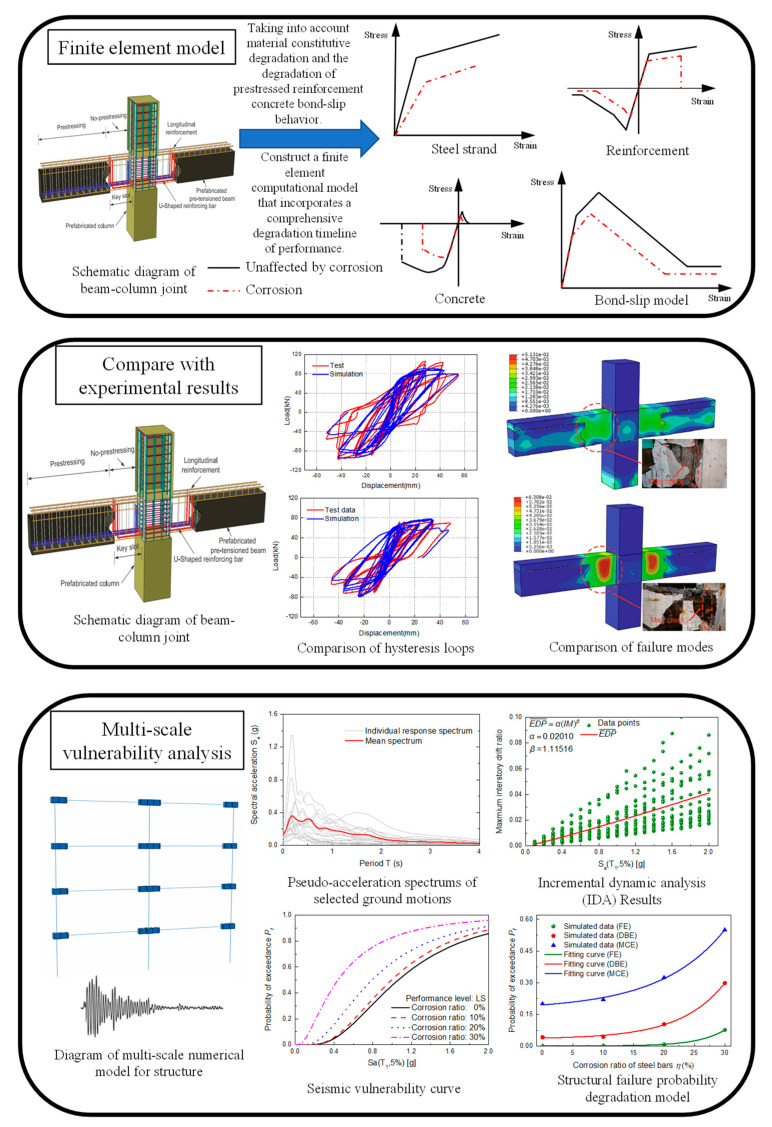
Multi-scale modeling method of “material–component–structure”.

To accurately conduct earthquake vulnerability analysis and comprehensively capture the structural response under seismic forces, it is imperative to establish precise and efficient numerical models. In pursuit of this objective, a multi-scale modeling method of “material–component–structure” is proposed, as illustrated in Figure 4 [11]. Structural corrosion induces degradation in the constitutive models for reinforcement, concrete, and the bond-slip between reinforcement and concrete. To develop accurate and efficient numerical models, it is advisable to engage in relevant research or refer to the existing literature to formulate constitutive models that incorporate the effects of corrosion on reinforcement, concrete, and the bond-slip between reinforcement and concrete. Once these constitutive models are established, corresponding numerical models should be constructed based on the actual structure. The numerical analysis results should be compared with experimental data to validate the accuracy of the modeling approach. Seismic vulnerability analysis demands extensive computational analysis. Therefore, while ensuring the accuracy of the analysis results, efforts should be made to maximize the computational efficiency of numerical analysis. The adoption of a multi-scale modeling approach can effectively address this concern. Specifically, for regions such as beam–column joints that are structurally unique, experience complex forces, and are particularly sensitive to corrosion effects, fine-grained modeling employing solid elements is recommended. Conversely, for non-sensitive regions such as beams and columns, beam elements can be used for modeling.

## 5. Service Life Prediction

### 5.1. Engineering Case Study

**Figure 5:**
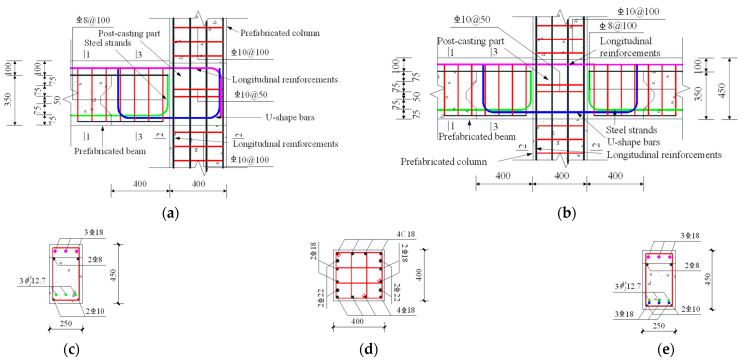
The dimensions and reinforcements of the frame structure. (**a**) External joint; (**b**) Interior joint; (**c**) Section 1; (**d**) Section 2; (**e**) Section 3.

This study selected a four-story precast prestressed concrete frame structure as the case study, as selected in the paper by Yang et al. [11]. The building was constructed with a seismic fortification intensity of 7 degrees (0.15 g), with seismic design grouped into the first category and a characteristic period of 0.35 s. The structure had a total length of 32.4 m and 12.0 m in the two plan directions, respectively, and a total height of 15.0 m. The floor live load was 4.5 kN/m^2^, and the roof floor live load was 7.0 kN/m^2^. The live load was taken as 2.0 kN/m^2^. The specific construction and reinforcement details are shown in Figure 5. The concrete strength of the precast beams and columns was C40, and the cast-in-place concrete strength was C45. The longitudinal bars in the beams and columns, as well as the U-shaped bars, were made of HRB400-grade steel. The stirrups were made of HRB335-grade steel.

### 5.2. Corrosion Initiation Stage

**Figure 6:**
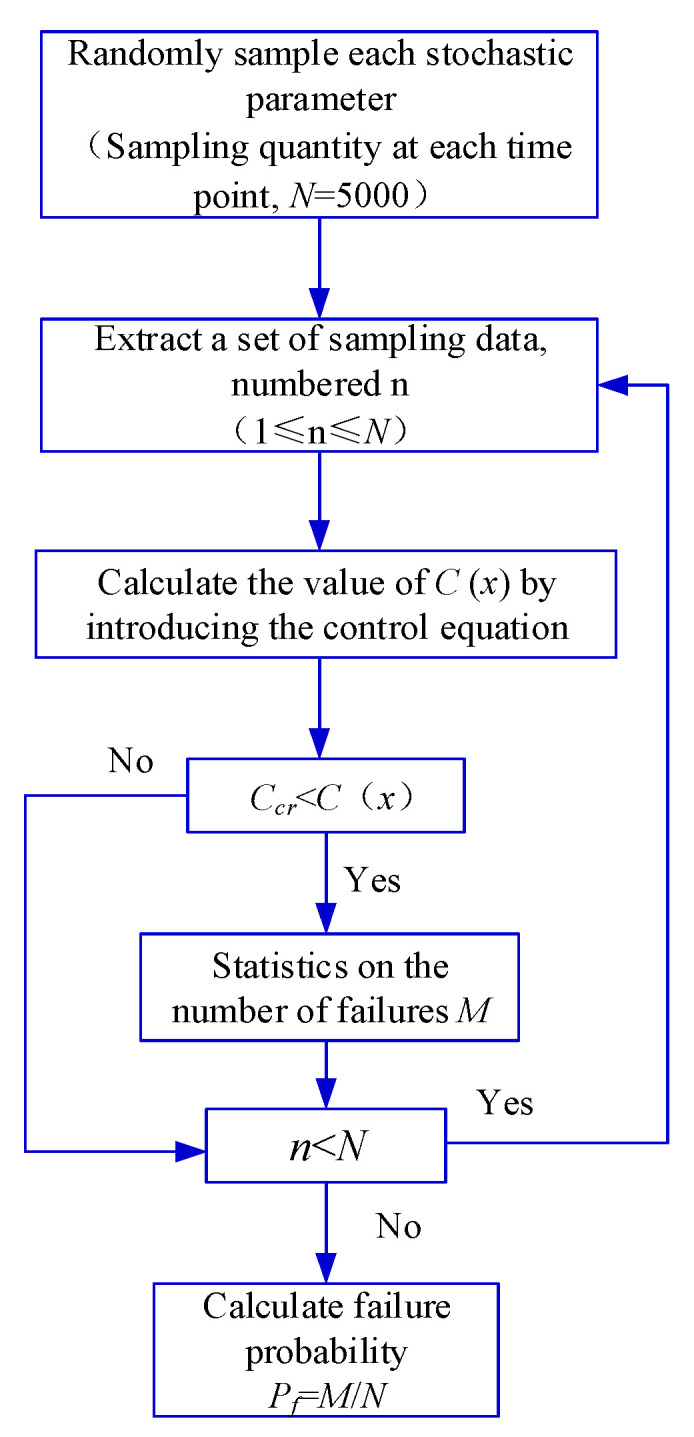
Procedure for calculating distribution characteristics of initial corrosion time.

**Figure 7:**
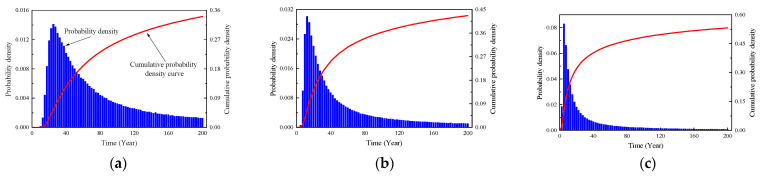
Probability distribution characteristics of initial corrosion time of steel bars in different parts. (**a**) Cast-in-site part; (**b**) Precast part; (**c**) Composite layer part.

Referring to the relevant literatures, the input parameters adopted in this study are shown in Table 2. It should be noted that the random distribution characteristics of the input parameters exhibit a certain degree of randomness and require a lot of statistical analysis, particularly in relation to the coefficient of the variation and the distribution pattern associated with these characteristics. In this study, the random distribution characteristics of the input parameters were determined based on the actual dimensions of the structure and referenced from similar literature. For differing conditions and regions, it is recommended to conduct an investigation to establish the random distribution characteristics of the input parameters. Based on Equation (9), the initial corrosion time distribution of the rebars was calculated using the Monte-Carlo simulation. The simulation was implemented using MATLAB software (version number: 2020a), and its computational process is shown in Figure 6. At each time point, 5000 samples were extracted, resulting in a total of 1,000,000 samples. The analysis results are shown in Figure 7. According to Figure 7, the time taken for the cast-in-place part, prefabricated part, and composite-layer part to reach the depassivation state was 33.4 years, 15.2 years, and 5.0 years, respectively.

### 5.3. Corrosion Progression Stage

**Figure 8:**
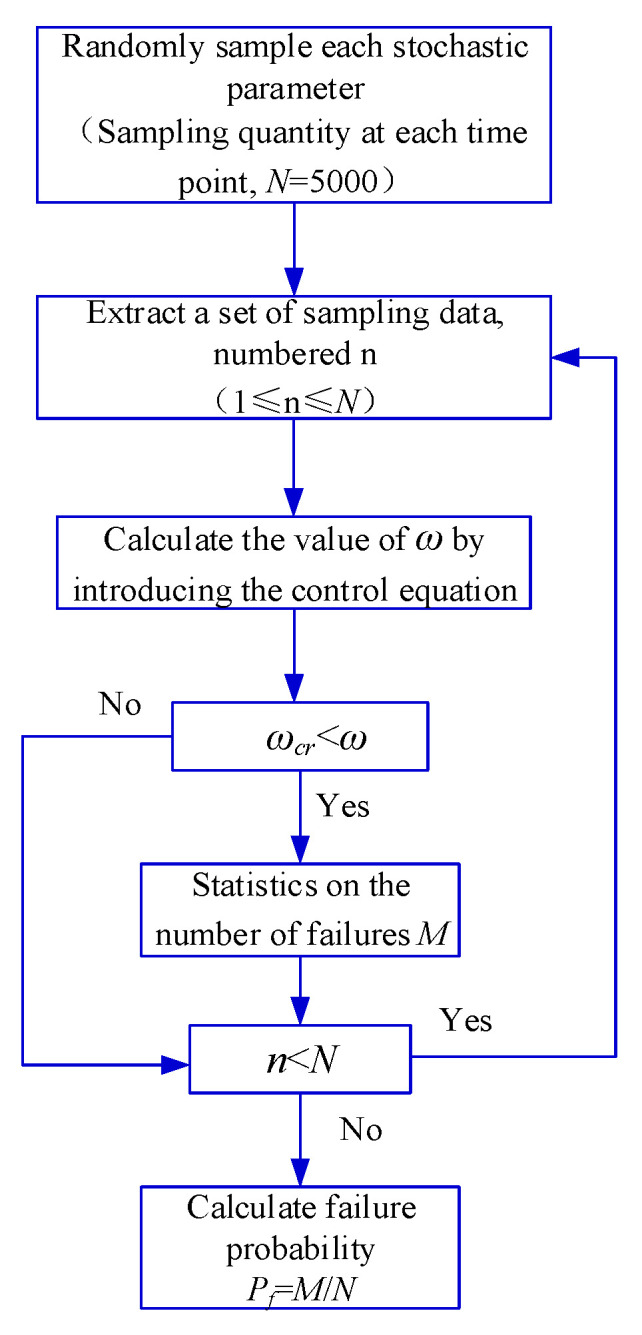
Procedure for calculating distribution characteristics of corrosion progression period time.

**Figure 9:**
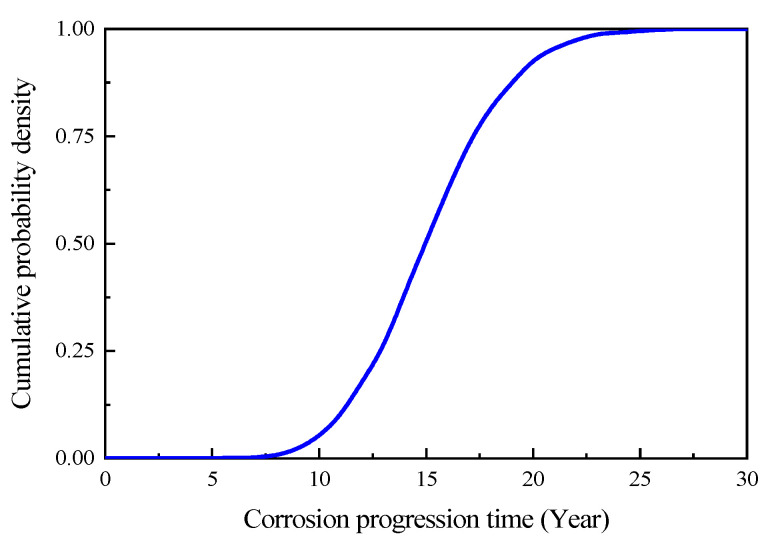
Cumulative probability density of a corrosion expansion crack reaching critical crack width.

In this study, a critical crack width of 0.2 mm was utilized as the endpoint for the corrosion progression stage. According to previous studies, the duration of the corrosion progression stage is relatively short compared to the entire service life of concrete structures. Typically, following the onset of the corrosion initiation stage, concrete spalling due to corrosion-induced expansion occurs within a short period of time. Therefore, in this study, Equation (14) was used for the calculation of the corrosion ratio during both the corrosion progression stage and corrosion failure stage. Statistical analysis was performed using Monte-Carlo simulation, and the simulation was implemented using MATLAB software, as illustrated in Figure 8. The sampling was performed 1,500,000 times, with 5000 samples at each time point. Additionally, the steel corrosion ratio (*η*) was determined using Equation (24). After entering the corrosion progression stage, the cumulative probability density curve of corrosion expansion cracks reaching the critical crack width is shown in Figure 9.

(24) $$\eta =\frac{\Delta A_{s}}{A_{0}}=\frac{4\Delta A_{s}}{\pi d_{s0}^{2}}$$

where *A*_0_ represents the original cross-sectional area of the uncorroded steel reinforcement.

### 5.4. Corrosion Failure Stage

**Figure 10:**
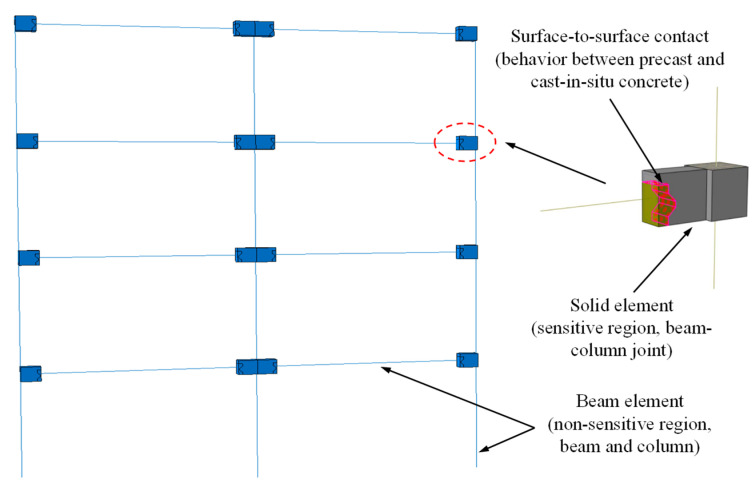
Schematic diagram of multi-scale model.

A schematic diagram of the multi-scale vulnerability analysis model is shown in Figure 10. In regions of high sensitivity, such as beam–column joints, solid elements were employed. Conversely, in non-sensitive regions such as beams and columns, beam elements were utilized for modeling. The interaction behavior between precast and cast-in-situ concrete was simulated through surface-to-surface contact in Abaqus, wherein a friction coefficient of 0.6 [43] was employed to replicate tangential behavior, and a stiffness scale factor of 1.0 was utilized to model normal behavior. A comparative analysis between the numerical simulation results and experimental findings was presented in reference [11]. According to the reference, this multi-scale model has demonstrated improved computational efficiency while maintaining accuracy. In this study, based on the multi-scale vulnerability analysis conducted by Yang et al. [11] for the structure shown in Figure 5, a probabilistic model for safety failure conditions during the corrosion failure period was developed. According to the research of Yang et al. [11], failure probability models for the structural performance variation with corrosion rate (*η*) under different performance level requirements were formulated, as shown in Equations (25)–(28). Here, FE denotes frequent earthquakes, DBE stands for design basis earthquakes, and MCE represents maximum credible earthquakes. In subsequent analyses, the reliability index of the structural ultimate limit state, under varying conditions, would be determined based on the failure probability models presented in Equations (25)–(28).

(25) $$\mathrm{OP}:\left\{\begin{array}{c} \mathrm{FE}:P_{f}=0.069+4.004\times 10^{-4}\times e^{\eta /4.178}\\ \mathrm{DBE}:P_{f}=0.573+8.466\times 10^{-4}\times e^{\eta /4.999}\\ \mathrm{MCE}:P_{f}=0.922+4.331\times 10^{-5}\times e^{\eta /4.119} \end{array}\right.$$

(26) $$\mathrm{IO}:\left\{\begin{array}{c} \mathrm{FE}:P_{f}=0.005+7.678\times 10^{-5}\times e^{\eta /3.681}\\ \mathrm{DBE}:P_{f}=0.175+0.795\times 10^{-2}\times e^{\eta /7.163}\;\\ \mathrm{MCE}:P_{f}=0.569+0.060\times e^{\eta /17.290} \end{array}\right.$$

(27) $$\mathrm{LS}:\left\{\begin{array}{c} \mathrm{FE}:P_{f}=-1.286\times 10^{-4}+1.286\times 10^{-4}\times e^{\eta /4.740}\\ \mathrm{DBE}:P_{f}=0.022+8.670\times 10^{-4}\times e^{\eta /5.005}\\ \mathrm{MCE}:P_{f}=0.145+0.078\times e^{\eta /15.197} \end{array}\right.$$

(28) $$\mathrm{CP}:\left\{\begin{array}{c} \mathrm{FE}:P_{f}=-1.690\times 10^{-4}+1.690\times 10^{-4}\times e^{\eta /5.459}\\ \mathrm{DBE}:P_{f}=0.010+2.296\times 10^{-4}\times e^{\eta /4.263}\\ \mathrm{MCE}:P_{f}=0.084+0.049\times e^{\eta /12.590} \end{array}\right.$$

### 5.5. Lifetime Seismic Performance Evaluation Based on the Path Probability Model

The degradation process of concrete structures in chloride environments exhibits distinct stages, and each stage has significantly different deterioration models. To improve analysis efficiency, Monte-Carlo simulation can be used to independently calculate the conditional probability of failure at each stage or the conditional probability distribution of each key parameter. Subsequently, the path probability model [20] is used to concatenate the calculation results of each stage and summarize them to form a time-varying reliability model of the structure in each limit state. According to the path probability model, when predicting the corrosion state of the structure at time *t*, the corrosion can only occur within the interval [0, *t*). Otherwise, the event probability is zero. By dividing the interval [0, *t*) into *n* sub-intervals, (n − 1) paths are formed. For each path, it is assumed that the corrosion initiation period ends at time *t_i_* and enters the corrosion progression period, with corrosion progressing over the remaining time. All paths are mutually exclusive, and according to the law of total probability, the contributions of each path can be summed to calculate the corrosion level distribution at time *t*.

Therefore, assuming *t_ini_* represents the moment when the corrosion initiation period ends (i.e., the onset of initial corrosion), the probability density of crack width *ω* at time *t* is given as following equation:

(29) $$f\left(\omega \right)=\sum_{i=1}^{n}f\left(\omega ,t_{ini}\right)=\sum_{i=1}^{n}f\left(\omega \left|t_{ini}\right.\right)p\left(t_{ini}\right)$$

where *p*(*t_ini_*) is the probability that the corrosion initiation stage (i.e., the initial rust begins to appear) would end exactly at the moment *t_ini_*.

Similarly, the corrosion ratio distribution at any given moment can be calculated using Equation (30).

(30) $$f\left(\eta \right)=\sum_{i=1}^{n}f\left(\eta ,t_{ini}\right)=\sum_{i=1}^{n}f\left(\eta \left|t_{ini}\right.\right)p\left(t_{ini}\right)$$

**Figure 11:**
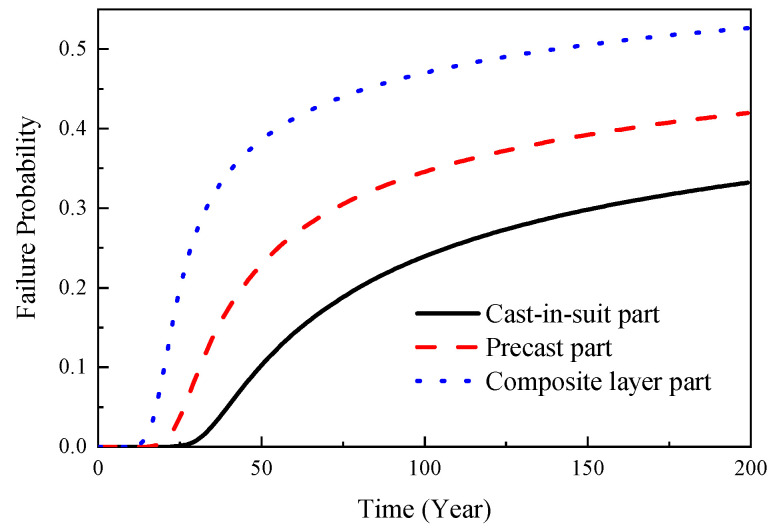
Time-dependent reliability model for normal serviceability limit state.

Based on Equation (30) and Figure 9, the time-dependent reliability model curves for the structure in a normal serviceability limit state could be obtained, as shown in Figure 11. According to Figure 11, the service life for the cast-in-place part, precast part, and composite layer part were determined to be 42.8 years, 27.9 years, and 18.7 years, respectively.

**Figure 12:**
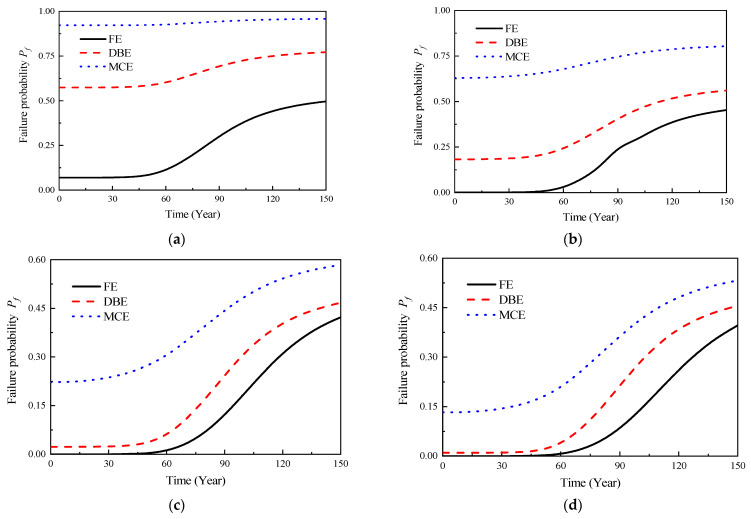
Time-dependent reliability model curves for different ultimate limit states (prefabricated frame). (**a**) OP; (**b**) IO; (**c**) LS; (**d**) CP.

**Figure 13:**
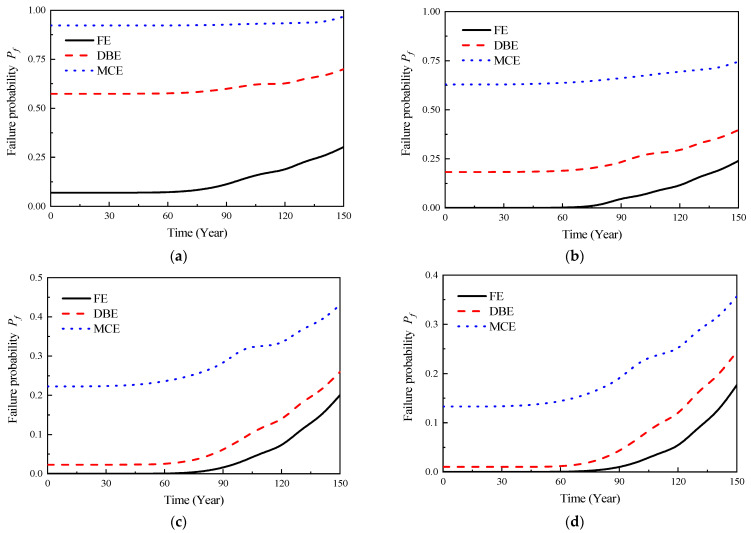
Time-dependent reliability model curves for different ultimate limit states (equivalent cast-in-place frame). (**a**) OP; (**b**) IO; (**c**) LS; (**d**) CP.

In accordance with Equation (30) and in conjunction with Figure 8 and Equations (25)–(28), the reliability model curves of the structural capacity limit state under various performance levels could be obtained, as shown in Figure 12 and Figure 13. As shown in Figure 7, it could be observed that the composite layer part, due to a higher chloride ion diffusion coefficient, led to an earlier initiation of corrosion in the reinforcement, gradually resulting in structural failure. Moreover, as the composite layer part was closer to the vulnerable regions of the precast prestressed concrete structure, the probability distribution characteristics of the initial corrosion time in the composite layer part were used as the basis to establish the time-dependent reliability model curves of the precast prestressed concrete structure, as shown in Figure 12. Simultaneously, using the probability distribution characteristics of the initial corrosion time in the cast-in-place part as a foundation, time-dependent reliability model curves of equivalent cast-in-place frames were established, as shown in Figure 13. It was evident from the figures that the seismic performance of the precast prestressed concrete structure gradually decreased with the development of corrosion. Comparing Figure 12 and Figure 13, it was apparent that the presence of the composite layer led to a more severe deterioration in the time-dependent seismic performance of the precast prestressed concrete structure compared to the equivalent cast-in-place structure. For instance, under the action of maximum credible earthquakes in 50 years, the failure probability of the precast prestressed concrete structure in the CP limit state was 0.176, whereas the corresponding failure probability for the equivalent cast-in-place structure in the CP limit state was 0.138. Similarly, under the action of maximum credible earthquakes in 50 years, the failure probability of the precast prestressed concrete structure in the CP limit state was 0.413, whereas the corresponding failure probability for the equivalent cast-in-place structure in the CP limit state was 0.224.

## 6. Conclusions

Based on reliability theory, this study established a framework for evaluating the lifetime performance of a precast prestressed concrete frame in chloride environments. By applying the path probability model, time-varying reliability models for predicting the three limit states of the precast prestressed concrete structure were proposed, and a method for predicting the service life of the structure in chloride environments was developed. An example of a four-story planar frame was analyzed and studied. Based on the analysis results, the following conclusions were drawn:

1. The initial corrosion time of reinforcement was highly sensitive to the chloride ion diffusion coefficient. In the composite layer between the precast part and the cast-in-place part, the higher chloride ion diffusion coefficient would lead to early corrosion in the precast prestressed concrete frame, consequently reducing the durability of the structural system.
2. Both the depassivation of reinforcement and the limit state of normal service was highly sensitive to the chloride ion diffusion coefficient in the composite layer. For structures adopting these two limit states as design failure criteria, it was crucial to focus on improving the waterproofing performance of the joint area to extend the service life of the structure.
3. Using the time-dependent reliability model curves of structural capacity limit states under different performance levels, it was observed that the seismic performance of the precast prestressed concrete frame structures gradually decreased with the development of corrosion. The presence of the composite layer led to a more severe deterioration in the time-dependent seismic performance of the precast prestressed concrete frame structure compared to the cast-in-place structure.

## Figures and Tables

**Table 1:** Target reliability indexes and failure probability of the durability limit states.

Durability Limit State	[*β*]	[*P_f_*]	Reference
Corrosion initiation	1.3	9.70 × 10^−2^	[24]
Corrosion-induced crack reaching the maximum acceptable level	1.5	6.68 × 10^−2^	[25]

**Table 2:** Random distribution characteristics of input parameters.

Input Parameter	Unit	Average Value	Coefficient of Variation	Distribution Pattern	Reference
Surface chloride ion concentration *C_s_*	%	0.13	0.7	Lognormal distribution	[40]
Critical chloride ion concentration *C_cr_*	%	0.12	0.6	Lognormal distribution	[34]
Chloride ion diffusion coefficient *D*_0_ (Cast-in-site part)	mm^2^/a	31.5	0.2	Lognormal distribution	[23]
Chloride ion diffusion coefficient *D*_0_ (Precast part)	mm^2^/a	63	0.2	Lognormal distribution	[23]
Chloride ion diffusion coefficient *D*_0_ (Composite layer part)	mm^2^/a	158	0.2	Lognormal distribution	[27]
Protective layer thickness *x* (Cast-in-situ part)	mm	50	0.05	Normal distribution	[34]
Protective layer thickness *x* (Precast part)	mm	67	0.05	Normal distribution	[34]
Protective layer thickness *x* (Composite layer part)	mm	50	0.05	Normal distribution	[34]
*n*	-	0.362	0.677	Beta distribution	[41,42]
*k_l_*	-	0.832	0.028	Normal distribution	[41,42]
*k_e_*	-	0.676	0.168	Gamma distribution	[41,42]
*k_e_*	-	1	-	-	[41,42]

## Data Availability

Data available on request due to restrictions eg privacy or ethical. The data presented in this study are available on request from the corresponding author. The data are not publicly available due to follow-up research on the work.
